# Revisiting *Leishmania* (*Viannia*) *lainsoni* Silveira, Shaw, Braga, and Ishikawa, 1987: current knowledge and emerging perspectives

**DOI:** 10.1186/s13071-026-07368-3

**Published:** 2026-03-25

**Authors:** Thais Gouvea de Morais, Marliane Batista Campos, Fernando Tobias Silveira, Thiago Vasconcelos dos Santos

**Affiliations:** 1 Programa de Pós-Graduação em Biologia de Agentes Infecciosos e Parasitários, Instituto de Ciências Biológicas, Universidade Federal do Pará, Belém, PA Brazil; 2 Laboratório de Leishmanioses “Dr. Ralph Lainson’’, Seção de Parasitologia, Instituto Evandro Chagas, Ananindeua, PA Brazil; 3 Departamento de Patologia, Faculdade de Medicina, Universidade de Sao Paulo, Sao Paulo, SP Brazil; 4 Programa de Pós-Graduação em Biologia Parasitária na Amazônia, Centro de Ciências Biológicas e da Saúde, Universidade do Estado do Pará, Belém, PA Brazil; 5 Programa de Pós-Graduação em Doenças Tropicais, Núcleo de Medicina Tropical, Universidade Federal do Pará, Belém, PA Brazil

**Keywords:** Biology, Divergence, Ecology, Emerging, *Leishmania* (*Viannia*) *lainsoni*, Phylogeny

## Abstract

More than three decades after its description, *Leishmania* (*Viannia*) *lainsoni* Silveira, Shaw, Braga, and Ishikawa, 1987 remains the least explored species of the subgenus *Viannia* Lainson and Shaw, 1987 within the genus *Leishmania* Ross, 1903. Although long considered rare and of limited clinical impact, recent records reveal a wider geographic range, diverse vector associations, and increasing involvement in American cutaneous leishmaniasis (ACL). Advances in molecular and genomic studies have clarified its taxonomy and uncovered notable biological and genetic variability. This review synthesizes current knowledge on the distribution, vectors, reservoirs, pathogenicity, and clinical relevance of *L*. (*V*.) *lainsoni*, highlighting emerging evidence that challenges earlier assumptions and emphasizing key research gaps for improving surveillance and understanding of its transmission dynamics.

**Graphical Abstract:**

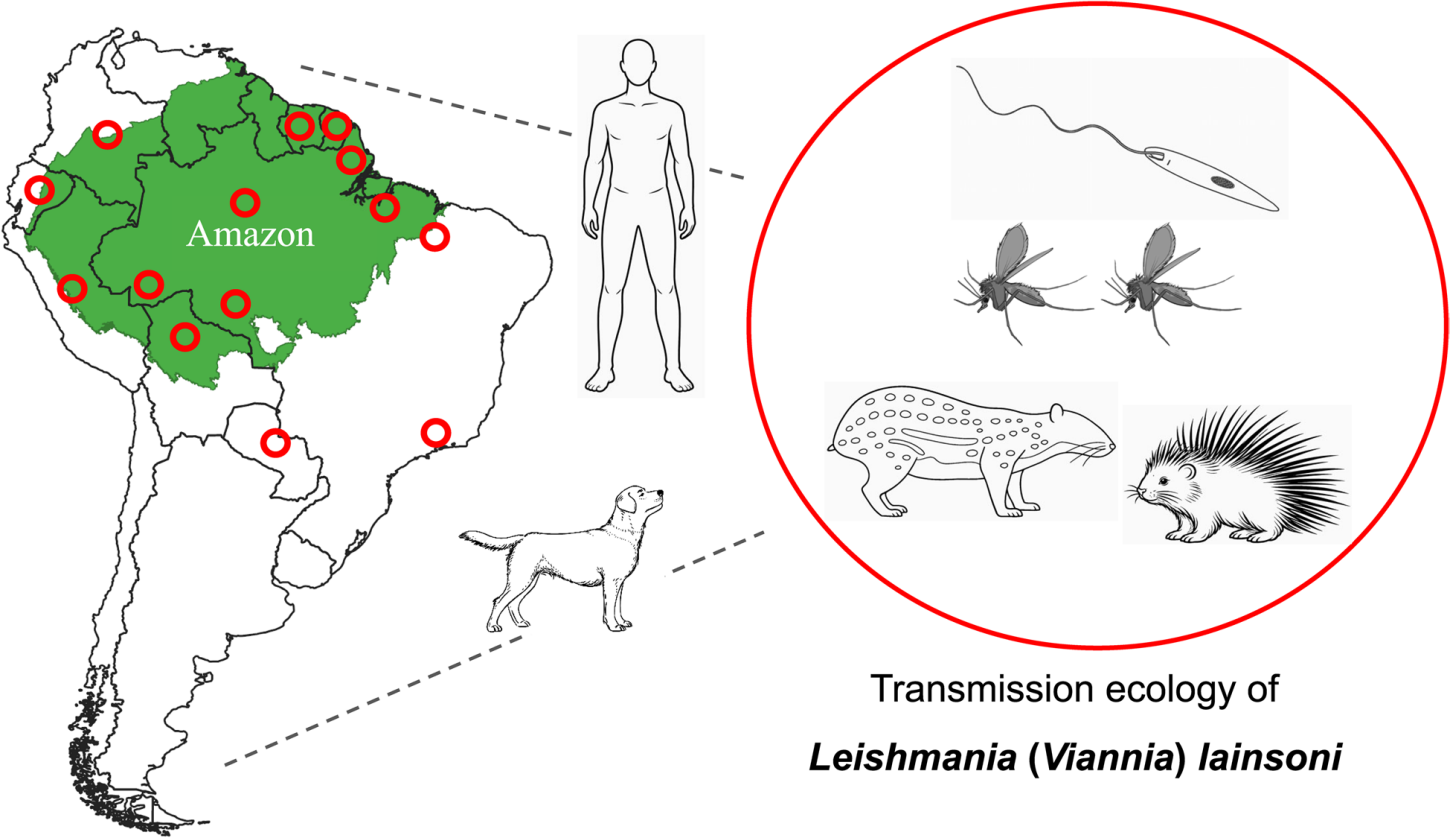

## Background

Within the genus *Leishmania* Ross, 1903 (Trypanosomatidae: Leishmaniinae), more than 20 species are recognized as etiological agents of human disease, classified into the subgenera *Viannia* Lainson and Shaw, 1987; *Leishmania* Saf'janova, 1982; and *Mundinia* Shaw, Camargo and Teixeira, 2016 [1, 2]. These parasites have a digenetic life cycle that involves both vertebrate and invertebrate hosts. Transmission occurs when an infected female sand fly inoculates metacyclic promastigotes during blood feeding. In the vertebrate host tissues, promastigotes are rapidly internalized by cells of the mononuclear phagocyte system, primarily macrophages, where they differentiate into amastigotes within phagolysosomes [3]. Approximately 100 sand fly species are estimated to act as vectors of *Leishmania* spp. [4], and the vertebrates most frequently implicated as reservoirs include dogs, edentates, primates, rodents, and marsupials [5].

Leishmaniasis is widely distributed across tropical and subtropical regions of Africa, America, Asia, and Europe [6], with an estimated 700,000 to 1 million new cases annually of cutaneous (CL) and visceral leishmaniasis (VL) [2]. In the Americas, 22 countries report leishmaniasis, with 19 being endemic for American cutaneous leishmaniasis (ACL) and 13 for American visceral leishmaniasis (AVL), and approximately 16 *Leishmania* spp. associated with human infection in the region [7, 8].

A wide spectrum of clinical manifestations is observed in ACL, depending on host genetic factors and the infecting species. These include localized cutaneous leishmaniasis (LCL), mucocutaneous leishmaniasis (ML), borderline disseminated cutaneous leishmaniasis (BDCL), and anergic diffuse cutaneous leishmaniasis (ADCL) [5]. Among the agents of ACL, *Leishmania* (*Viannia*) *lainsoni* Silveira, Shaw, Braga, and Ishikawa, 1987 is noteworthy for exhibiting traits that diverge from other species in the subgenus *Viannia* [9]. This species was described from isolates obtained from six patients presenting single cutaneous lesions in Pará, Brazil. Its distinctiveness was demonstrated through morphological, biochemical, and serological analyses. Morphologically, the parasites exhibited elongated, nearly fusiform amastigotes (mean length 3.49 µm; mean width 1.40 µm), and promastigotes with a mean body length of 17.43 µm and a flagellum length of 25.70 µm; values exceeding those reported for all other known species of the subgenus *Viannia*, even greater than that of *Leishmania* (*Leishmania*) *amazonensis* Lainson and Shaw, 1972 (11.86 µm and 14.03 µm, respectively) [F.T. Silveira, personal observation]. Enzymatic profiling differentiated the species by six of the ten tested enzymes, and none of the evaluated monoclonal antibodies reacted with the isolates, confirming their uniqueness [10].

Since its description, the parasite has been reported from *Trichophoromyia* Barretto, 1962 sand flies [11], wild mammals [12], and numerous human cases throughout South America [14–23]. Nonetheless, the true geographical range of the leishmanial species, as well as its vectors and reservoir hosts, is likely underestimated.

This review aims to synthesize current knowledge on the eco-epidemiological, biological, and phylogenetic features of *L*. (*V*.) *lainsoni*, and to identify critical knowledge gaps that should guide future investigations.

### Geographic distribution of human cases

**Fig. 1:**
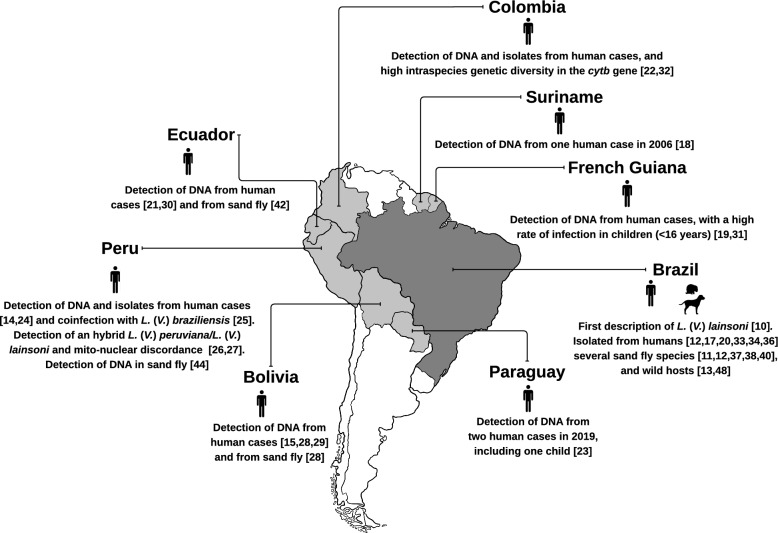
Geographic distribution and epidemiological evidence of *Leishmania* (*Viannia*) *lainsoni* in South American countries. *DNA:* deoxyribonucleic acid, *cytb:* cytochrome b, *L*.: *Leishmania, V*.: *Viannia*

Several countries in the Amazon region have reported ACL caused by this species. In Peru, multiple cases have been confirmed [14, 24], including one co-infection with *L*. (*V*.) *braziliensis* Vianna, 1911 [25]. An isolate exhibiting disagreement between mitochondrial and nuclear genetic markers has also been described, as well as a possible *L*. (*V*.) *peruviana* Velez, 1913/*L*. (*V*.) *lainsoni* hybrid parasite [26, 27]. Additional human ACL cases have been recorded in Bolivia [15, 28, 29], Suriname [18], French Guiana [19], and the Amazon region of Ecuador [21, 30], where high case frequency has been observed among children younger than 16 years [31]. In Colombia, human cases have been reported [22], and two isolates obtained from geographically distant patients showed marked genetic divergence in the cytochrome b gene [32]. Two human cases have also been reported in Paraguay, including one in a 5-year-old child [23] (Fig. 1).

The first records of ACL by *L*. (*V*.) *lainsoni*, which supported its original description, were recorded in Pará State, Brazil, from patients presenting LCL [10]. Additional studies later confirmed this species as a causative agent of LCL in the same region [33]. A retrospective survey from 1995 to 2018 identified *L*. (*V*.) *lainsoni* as the second most frequent etiological agent of LCL in the metropolitan region of Belém, indicating a substantial presence in periurban environments [34].

**Fig. 2:**
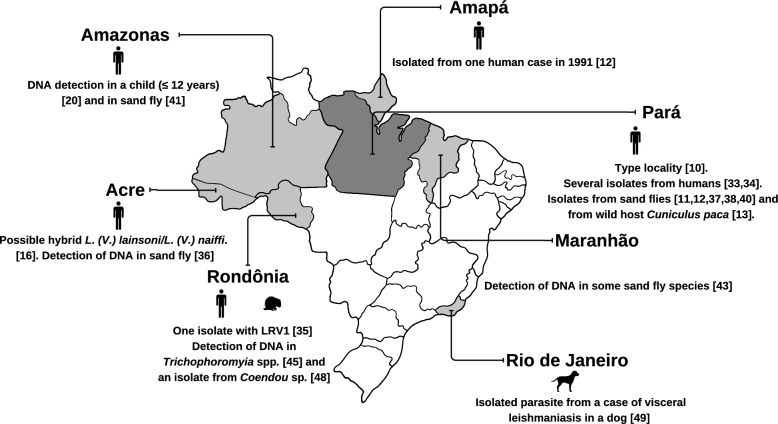
Geographic distribution and epidemiological evidence of *Leishmania* (*Viannia*) *lainsoni* in states and regions of Brazil. *DNA:* deoxyribonucleic acid, *L*.: *Leishmania, V*.: *Viannia*, *LRV1: Leishmania* RNA virus 1

Human ACL cases have also been documented in other states of the Brazilian Amazon. These include Amapá [12], Rondônia [17], where one isolate carried *Leishmania* RNA virus 1 (LRV1) [35], and Amazonas, where a pediatric case in a patient aged 12 years or younger was reported [20]. In Acre, a possible hybrid parasite between *L*. (*V*.) *lainsoni* and *L*. (*V*.) *naiffi* Lainson and Shaw, 1989 was detected [16], along with a more recent record suggesting urban transmission [36] (Fig. 2).

### Vectors

The first isolation of *L*. (*V*.) *lainsoni* from a sand fly occurred in 1983 in the Serra dos Carajás, Pará State, Brazil, in *Trichophoromyia ubiquitalis* (Mangabeira, 1942). At that time, the parasite was classified as an unnamed member of the subgenus *Viannia* [37], and it was later formally described as *L*. (*V*.) *lainsoni* [12]. Some phlebotomine sand fly species of the genus *Trichophoromyia* have been associated with this parasite, including *Th*. *ubiquitalis*, *Th*. *brachipyga*, and *Th*. *auraensis* (Mangabeira, 1942) [11].

*Leishmania* (*V*.) *lainsoni* promastigotes were again isolated from *Th*. *ubiquitalis* in the Utinga Park, the Belém metropolitan region, Pará State. Among the 375 specimens collected, eight of the nine infected sand flies carried *L*. (*V*.) *lainsoni* [12]. In another investigation, new isolates were obtained from *Th*. *ubiquitalis*, which was the only infected species among ten sand fly species captured in a primary forest area with ACL transmission. The same study reported an additional infected specimen of *Th*. *ubiquitalis* in Carajás, Pará State [38].

Despite multiple findings of natural infection in *Th*. *ubiquitalis*, this sand fly species is considered to have low affinity for feeding on humans in forested environments and is thought to bite humans only under specific conditions [38]. Nonetheless, in an experimental setting, 71 of 83 specimens were fed to a human volunteer 48 h after being captured in a forested environment [12]. Shaw [39] suggests that available evidence indicates that some species of *Trichophoromyia* may act as accidental human biters among vectors of ACL agents.

*Trichophoromyia brachipyga* has also been proposed as a suspected vector of *L*. (*V*.) *lainsoni* on the basis of findings of natural infection. During a field study conducted in an urban park in Belém, Pará State, Sánchez-Uzcátegui et al. [40] isolated the parasite from that species. Although *Th*. *ubiquitalis* was the most abundant sand fly in that study, no infection was detected in that species. Follow-up investigations revealed infections in two *Th*. *brachipyga* and four *Th*. *ubiquitalis* sand flies in Serra dos Carajás [11].

Beyond parasite isolation, molecular analyses have detected *L*. (*V*.) *lainsoni* DNA in *Th*. *ubiquitalis* in Amazonas State, Brazil [41], and in Ecuador [42]. In the Maranhão State, northeastern Brazil, the parasite’s DNA was found in *Evandromyia evandroi* (Costa Lima and Antunes, 1936), *Lutzomyia longipalpis* (Lutz and Neiva, 1912), and *Nyssomyia whitmani* (Antunes and Coutinho, 1939) [43]. In the state of Acre, Brazil, the parasite DNA was detected in *Bichromomyia flaviscutellata* (Mangabeira, 1942) [36]. Molecular evidence of *L*. (*V*.) *lainsoni* infection has also implicated *Th*. *auraensis* in Peru [44] and *Pintomyia nuneztovari* (Ortiz, 1954) in Bolivia [28]. More recently, DNA consistent with *L*. (*V*.) *lainsoni* was also detected in *Trichophoromyia* sand flies in Rondônia State, Brazil, although species-level identification was not possible [45] (Table 1). These findings suggest that the sand fly species involved in the transmission of *L*. (*V*.) *lainsoni* may vary across geographic locations.

**Table 1:** Detection of *Leishmania* (*Viannia*) *lainsoni* in different sand fly species in South American countries

Sand fly species	Location	Detection type	References
*Trichophoromyia ubiquitalis*	BRA, ECU	Parasite isolate, detection of DNA	[12, 42]
*Trichophoromyia brachipyga*	BRA	Parasite isolate	[40]
*Trichophoromyia auraensis*	PER	Detection of DNA	[44]
*Trichophoromyia* spp.	BRA	Detection of DNA	[45]
*Evandromyia evandroi*	BRA	Detection of DNA	[43]
*Lutzomyia longipalpis*	BRA	Detection of DNA	[43]
*Nyssomyia whitmani*	BRA	Detection of DNA	[43]
*Bichromomyia flaviscutellata*	BRA	Detection of DNA	[36]
*Pintomyia nuneztovari*	BOL	Detection of DNA	[28]

*BRA:* Brazil, *ECU:* Ecuador, *PER:* Peru, *BOL:* Bolivia, *DNA:* deoxyribonucleic acid, *spp*.: species

Experimental studies conducted both in vivo and in vitro have demonstrated that *L*. (*V*.) *lainsoni* can infect and develop to the metacyclic stage in *Lu*. *longipalpis* and *Ny. antunesi* Coutinho, 1939 [46], and can strongly adhere to the midgut of *Th*. *ubiquitalis*, *Th*. *brachipyga*, and *Lu*. *longipalpis* [47]. Collectively, these observations support the potential involvement of *Trichophoromyia* spp. and other sand flies in the transmission cycle.

### Reservoir hosts

The wild reservoir of *L*. (*V*.) *lainsoni* was first identified in Pará, in the lowland paca, *Cuniculus paca* (Linnaeus, 1766) (Rodentia: Cuniculidae), on the basis of the isolation of parasites from the skin of this animal with no apparent lesions. Although amastigotes were not observed on direct microscopy, inoculation of skin fragments into a hamster paw led to lesion development and detection of amastigotes after 3 months, with no indication of visceral dissemination. Species identification relied on promastigote and amastigote morphology, infection dynamics in golden hamsters, *Mesocricetus auratus* Waterhouse, 1839 (Rodentia: Cricetidae), enzymatic profiling, and monoclonal antibody reactivity [13]. The parasite was later reported in a porcupine of the genus *Coendou* Lacépède, 1799 (Rodentia: Erethizontidae) [48], originating from an isolation made in 1983 in Rondônia. The isolate was deposited in the Coleção de *Leishmania* da Fiocruz (CLIOC) by Toby Barrett under the international code MCOE/BR/1983/IM1367 (IOC/L1058) [E. Cupolillo, personal communication]. To date, these represent the only confirmed detections of *L*. (*V*.) *lainsoni* in wild mammals.

A recent autochthonous case of canine VL in Rio de Janeiro State, Brazil, was attributed to *L*. (*V*.) *lainsoni* [49]. Although the diagnosis was well supported, comprehensive eco-epidemiological investigations are still lacking, preventing a clear assessment of the actual role of this species in the transmission cycle in the region. The detection of *L*. (*V*.) *lainsoni* far outside its well-established Amazonian range may suggest the existence of previously unrecognized transmission circuits; however, it may also reflect an incidental or fortuitous finding without sustained epidemiological relevance. This latter interpretation is supported by the long-standing characterization of *L*. (*V*.) *lainsoni* as a strictly dermotropic parasite in its native Amazonian context, with no consistent evidence of visceralization or stable involvement in zoonotic VL cycles.

### Diagnostic potential

Another key characteristic of *L*. (*V*.) *lainsoni* is its easy cultivation in different culture media and its high cell mass yield. In addition, *L*. (*V*.) *lainsoni* has proven to be an excellent antigenic alternative for diagnosing leishmaniasis. It was demonstrated that this species’ promastigote antigen, when used in serological diagnosis via indirect fluorescent antibody test (IFAT), is more sensitive than a commercial kit [50]; additionally, its axenic amastigote antigen demonstrates sensitivity comparable to that of *L*. (*L*.) *amazonensis* [51]. More recently, it has also been demonstrated that its axenic amastigote antigen is more reactive than the promastigote antigen of *L*. (*V*.) *braziliensis* for the diagnosis of ACL using the delayed-type hypersensitivity reaction (Montenegro skin test) [52]. This highlights the potential of this species’ antigen in laboratory tests for ACL diagnosis.

### Drug susceptibility

The susceptibility of *L*. (*V*.) *lainsoni* to meglumine antimoniate, amphotericin B, or miltefosine was evaluated using Bolivian isolates. The parasites demonstrated resistance to meglumine antimoniate at both the promastigote and amastigote stages, even at the maximum concentration. In contrast, isolates of this species were sensitive to amphotericin B and miltefosine in both stages, with the amastigote stage less susceptible to miltefosine than the promastigote stage [29]. These results indicate that *L*. (*V*.) *lainsoni* may exhibit resistance to some conventional treatments, especially in the amastigote stage, a finding which may influence therapeutic choices and corroborates the importance of species-level diagnosis, particularly in areas where it has been reported to circulate.

Conversely, this does not reflect our clinical experience in treating ACL caused by *L*. (*V*.) *lainsoni* at the leishmaniasis outpatient clinic of the Evandro Chagas Institute in Pará State, Brazil, where treatment with meglumine antimoniate has been uniformly successful, with no documented relapses [F.T. Silveira, personal observation].

### Immune evasion mechanisms

Some interesting cellular characteristics and immune evasion mechanisms of *L*. (*V*.) *lainsoni* have been documented. The occurrence of promastigote forms within cellular vacuoles was reported in skin biopsies from hamsters infected with this species at 45 days post-infection. In vitro, these forms were also observed in J774-G8 macrophages 24 h after infection, and occasionally after 48 h post-infection. An interesting aspect of these results, as noted by Correa and Soares [53], is that intravacuolar amastigotes may give rise to promastigote forms; however, no mechanistic explanation for this observation is available. Subsequently, the same authors documented approximately 40% of amastigotes residing within free parasitophorous vacuoles. These vacuoles indicated the degradation and rupture of the host cell membrane, while the intact vacuole protected the parasite. This characteristic may suggest an evasion mechanism in this species, since phagocytosis of these vacuoles by macrophages would not activate the cellular microbicidal cascade [54].

### Pathogenicity and virulence

Infections by *L*. (*V*.) *lainsoni* have yielded interesting results in both in vitro and in vivo studies. In peritoneal BALB/c mice macrophages, *L*. (*V*.) *lainsoni* induced significantly higher nitric oxide (NO) production compared with isolates of *L*. (*V*.) *guyanensis* Floch, 1954 and *L*. (*V*.) *braziliensis* obtained from patients with mucosal leishmaniasis. Under these conditions, the macrophage infection index for *L*. (*V*.) *lainsoni* was significantly lower than that observed for the other isolates [55].

However, Soares et al. [56], in a recent study of infection in golden hamsters, *M. auratus*, described a high parasite load and intense inflammatory infiltrate for up to 40 days post-infection with *L*. (*V*.) *lainsoni*, similarly to *L*. (*V*.) *braziliensis* and *L*. (*V*.) *guyanensis*. In the same study, in vitro infection of hamster peritoneal macrophages and THP-1 cells with *L*. (*V*.) *lainsoni* was moderate. Furthermore, polymorphism was observed in the lipophosphoglycan (LPG) of *L*. (*V*.) *lainsoni*. Unlike all other species of *L*. (*Viannia*), the LPG of this species has a hexose side chain and induced greater production of NO, interleukin-6 (IL-6), and tumor necrosis factor alpha (TNF-α). Thus, although *L*. (*V*.) *lainsoni* can induce greater production of NO and proinflammatory cytokines under certain conditions, this does not appear to significantly compromise its ability to infect in a murine model initially.

However, this observation can be explained by the balance between proinflammatory and anti-inflammatory immune responses. Another in vivo study involving hamster paw and lymph node infection showed that, despite a high initial parasite load, the overall infection profile of *L*. (*V*.) *lainsoni* was intermediate. In the same study, the gene expression profile revealed high levels of inducible nitric oxide synthase (iNOS) and arginase in the hamster paws. Conversely, an increase in interleukin-12 (IL-12) and interleukin-10 (IL-10) expression was observed in the lymph nodes, along with a high overall expression of interferon gamma (IFN-γ). This may explain the initial difficulty in controlling the infection, followed by favorable immune modulation that reduces the parasite load at later times [57].

When infecting the tufted capuchin, *Sapajus apella* (Linnaeus, 1758) with *L*. (*V*.) *lainsoni*, Silveira et al. [58] observed that this primate developed lesions with a high amastigote load 30 days after infection. Most cases progressed to partial cure by 90 days, with complete cure by 120 days post-infection, a finding that reinforces the immune response profile discussed previously. In a subsequent comparison of infection in this animal model with *L*. (*L*.) *amazonensis*, *L*. (*V*.) *lainsoni*, and *L*. (*V*.) *braziliensis*, these authors determined that the lesions caused by the first two species, although from different subgenera, exhibited similar development and lacked ulceration. Conversely, *L*. (*V*.) *braziliensis* infection resulted in more extensive ulceration and necrosis, as well as a longer lesion evolution time [59].

### Distinctive features

Since its first description, multiple independent phenotypic and molecular approaches have consistently indicated that *L*. (*V*.) *lainsoni* differs from other species of the subgenus *Viannia*, despite its formal taxonomic placement within this group [1, 10]. Isoenzyme analysis showed that *L*. (*V*.) *lainsoni* exhibited a unique, highly distinct enzymatic profile, failing to group with any other *L*. (*Viannia*) spp., suggesting an independent evolutionary lineage within the subgenus [60]. It is assumed that *L*. (*V*.) *lainsoni* does not react with monoclonal antibodies (MAb) typical of its subgenus [10], just with the general trypanosomatid L1 [33] and occasionally LA2 [61, 62]. Corroborating these observations, a principal component analysis (PCA) based on biological, biochemical, and immunological characteristics of *L*. (*Viannia*) species identified in Brazil revealed the formation of two distinct clusters: one exclusive to *L*. (*V*.) *lainsoni* and the other comprising all remaining species, underscoring its divergence from the species of the same subgenus [56].

Molecular approaches targeting distinct genomic regions further reinforced this pattern of divergence. Literature frequently observes that *L*. (*V*.) *lainsoni* occupies a unique phylogenetic position, distant from other *L*. (*Viannia*) spp. [63]. It can be considered a divergent and monophyletic species, and even an independent complex within the subgenus *Viannia* [64]. In the comparison of genomic DNA digested with the restriction enzyme BamHI and hybridized with a radioactive β-tubulin gene probe, *L*. (*V*.) *lainsoni* exhibited a pattern very similar to that of species of the same subgenus, but distinct from *L*. (*Leishmania*) spp. Conversely, amplification of the kinetoplast DNA (kDNA) of this species resulted in a smaller fragment than that observed in other species of the same subgenus. Furthermore, kDNA digestion with HaeIII revealed a pattern distinct from other members of the subgenus *Viannia*, showing some similarity to the patterns observed in *L*. (*L*.) *amazonensis* and *L*. (*L*.) *mexicana* Biagi, 1953. However, the labeled kDNA of this species showed strong cross-hybridization not only with *L*. (*V*.) *braziliensis*, *L*. (*V*.) *naiffi*, and *L*. (*V*.) *shawi* Lainson, Souza, Póvoa, Ishikawa, and Silveira, 1989, but also with *L*. (*L*.) *amazonensis* and weakly with *L*. (*L*.) *mexicana* [63].

These observations led to more targeted molecular investigations. The finding that the kDNA minicircle region in *L*. (*V*.) *lainsoni* differs significantly from other members of the subgenus *Viannia* was initially suggested by the generation of unusually small PCR products using the universal primers B1 and B2 at lower-stringency annealing temperatures (60.5 °C), and by the absence of amplification at higher temperatures [63]. Following this observation, McCann et al. [65] sequenced the region and detected differences in the area adjacent to the conserved CSB3 block. This allowed the design of the specific primer SL3, which, when combined with B1, successfully amplified the complete minicircle exclusively in this species. Comparative sequencing analysis subsequently confirmed the phylogenetic proximity of *L*. (*V*.) *lainsoni* to species of *L*. (*Viannia*) spp., but also revealed molecular differences that underscore its taxonomic uniqueness within this group.

Independent nuclear markers have consistently reinforced this divergent genetic profile. Analyses of the *gp63*-encoding gene using gp63-restriction fragment length polymorphism (RFLP) and PCR–RFLP demonstrated that *L*. (*V*.) *braziliensis*, *L*. (*V*.) *peruviana*, and *L*. (*V*.) *guyanensis* are genetically closely related, whereas *L*. (*V*.) *lainsoni* is more distantly related, supporting its singular position within the subgenus *Viannia* [66]. In agreement with these findings, analysis of the ribosomal DNA internal transcribed spacer (ITS) region using intergenic region typing (IRT) revealed that *L*. (*V*.) *lainsoni* represents a clearly distinct lineage from other *L*. (*Viannia*) spp. [60]. Likewise, sequence analysis of a fragment of the *hsp70* gene from multiple *L*. (*Viannia*) spp. and *L*. (*Leishmania*) spp. isolates showed that *L*. (*V*.) *lainsoni* consistently forms a well-supported, genetically distinct clade within the subgenus *Viannia*, with low bootstrap support [67].

These observations were further corroborated by multilocus approaches. Multilocus sequence analysis (MLSA) of the glucose-6-phosphate dehydrogenase (*G6PD*), 6-phosphogluconate dehydrogenase (*6PGD*), mannose phosphate isomerase (*MPI*), and isocitrate dehydrogenase (*ICD*) genes identified *L*. (*V*.) *lainsoni* as the most genetically divergent species among members of the subgenus *Viannia*, together with *L*. (*V*.) *naiffi*, and additionally revealed high levels of intraspecific genetic diversity [68]. Consistently, multilocus microsatellite typing (MLMT) using 15 highly polymorphic microsatellite markers showed that *L*. (*V*.) *lainsoni* isolates display substantial genetic diversity, comparable to that observed in *L*. (*V*.) *shawi* and *L*. (*V*.) *naiffi*. In NeighborNet analyses, *L*. (*V*.) *lainsoni* clustered as a small, well-separated group, reinforcing its status as a distinct evolutionary lineage within the subgenus *Viannia* [69].

Finally, a preliminary genomic sequence of an *L*. (*V*.) *lainsoni* isolate from Peru was published in 2019, containing 140 contigs, 34,156,530 base pairs (bp), an N50 of 638,860 bp, and a G + C content of 57.8% [70]. Subsequently, a comparative study analyzed the proteomes of *L*. (*M*.) *orientalis* Bates and Jariyapan, 2018 and *L*. (*M*.) *martiniquensis* Desbois, Pratlong, and Dedet, 2014, comparing them with those of other *Leishmania* spp., including *L*. (*V*.) *lainsoni*. The results demonstrated that *L*. (*V*.) *lainsoni* exhibits numerous conserved proteins relative to other species of the genus, along with point variations that reflect its own molecular characteristics. In phylogenetic analysis based on protein sequence similarity, *L*. (*V*.) *lainsoni* clustered with *L*. (*V*.) *guyanensis*, while remaining distinct from *L*. (*Leishmania*) spp. [71].

Molecular clock analyses of the DNA polymerase-α subunit gene of *Leishmania* (*Viannia*) spp., including *L*. (*V*.) *lainsoni* (Peru), *L*. (*V*.) *peruviana* (Peru), *L*. (*V*.) *braziliensis* (Brazil), *L*. (*V*.) *guyanensis* (Brazil), and *L*. (*V*.) *panamensis* (Panama), indicate that *L*. (*V*.) *lainsoni* is the most ancient lineage within the subgenus [72].

## Conclusions

Collectively, these studies demonstrate that the geographic distribution of this species may be underestimated, since cases reported in areas relatively distant from the Amazon region, where it was first detected, suggest the emergence of new transmission cycles involving additional reservoirs and sand fly vectors. Moreover, the parasite’s biological characteristics underscore its relevance for scientific research aimed at elucidating *Leishmania*–host interaction mechanisms and support its potential use in diagnostic tool development. Furthermore, biochemical, immunological, and molecular evidence reinforces *L*. (*V*.) *lainsoni’s* unique position within the subgenus *Viannia*. Despite genetic characteristics that support its inclusion in this subgenus, diverse analyses, techniques, and sample sizes converge on the finding that this species occupies an isolated, divergent phylogenetic position. It may represent an independent evolutionary group, or even a specific complex, within the subgenus *Viannia*. Thus, *L*. (*V*.) *lainsoni* stands out as a relevant species for understanding the diversity and evolution of the genus *Leishmania*, and further genomic studies are needed to clarify its evolutionary relationship with other *L*. (*Viannia*) spp.

## Data Availability

All the data supporting the conclusions are included in the article.

## Abbreviations

*(L*.): **subgenus** Leishmania
ACL: American cutaneous leishmaniasis
*(V*.): *subgenus Viannia*
*(M*.): *subgenus Mundinia*
spp.: Species
CL: Cutaneous leishmaniasis
VL: Visceral leishmaniasis
AVL: American visceral leishmaniasis
LCL: Localized cutaneous leishmaniasis
ML: Mucocutaneous leishmaniasis
BDCL: Borderline disseminated cutaneous leishmaniasis
ADCL: Anergic diffuse cutaneous leishmaniasis
µm: Micrometers
LRV1: *Leishmania* RNA virus 1
RNA: Ribonucleic acid
*Th*.: *Trichophoromyia*
DNA: Deoxyribonucleic acid
*Lu*.: *Lutzomyia*
IFAT: Indirect fluorescent antibody test
NO: Nitric oxide
*M*.: *Mesocricetus*
THP-1: Tohoku Hospital Pediatrics-1
LPG: Lipophosphoglycan
IL-6: Interleukin-6
TNF-α: Tumor necrosis factor alpha
iNOS: Inducible nitric oxide synthase
IL-12: Interleukin-12
IL-10: Interleukin-10
IFN-γ: Interferon gamma
MAb: Monoclonal antibodies
PCA: Principal component analysis
kDNA: Kinetoplast deoxyribonucleic acid
PCR: Polymerase chain reaction
gp63: Glycoprotein 63
RFLP: Restriction fragment length polymorphism
ITS: Internal transcribed spacer
IRT: Intergenic region typing
MLSA: Multilocus sequence analysis
*G6PD*: Glucose-6-phosphate dehydrogenase
*6PGD*: 6-Phosphogluconate dehydrogenase
*MPI*: Mannose phosphate isomerase
*ICD*: Isocitrate dehydrogenase
MLMT: Multilocus microsatellite typing
